# Capillary-Bridge Controlled Patterning of Stable Double-Perovskite Microwire Arrays for Non-toxic Photodetectors

**DOI:** 10.3389/fchem.2020.00632

**Published:** 2020-08-25

**Authors:** Yueyang Pi, Jinjin Zhao, Yingjie Zhao, Jiangang Feng, Chi Zhang, Hanfei Gao, Yuchen Wu, Lei Jiang

**Affiliations:** ^1^Key Laboratory of Advanced Materials of Ministry of Education, School of Materials Science and Engineering, Tsinghua University, Beijing, China; ^2^Key Laboratory of Bio-Inspired Materials and Interfacial Science, Technical Institute of Physics and Chemistry, Chinese Academy of Sciences, Beijing, China; ^3^University of Chinese Academy of Sciences (UCAS), Beijing, China; ^4^Division of Physics and Applied Physics, School of Physical and Mathematical Sciences, Nanyang Technological University, Singapore, Singapore; ^5^Key Laboratory of Bio-Inspired Smart Interfacial Science and Technology of Ministry of Education, School of Chemistry, Beihang University, Beijing, China

**Keywords:** double perovskite, microwire array, photodetector, non-toxic, stable

## Abstract

Single-crystalline lead halide perovskites with remarkable physical properties offer great potential in integrated optoelectronic applications but are restricted by their instability and toxicity. To address these problems, various strategies including lead-free halide double perovskites with high stabilities of heat, light, and moisture have been developed. However, it still requires an efficient method to pattern single-crystalline, double-perovskite micro-/nanostructures with strict alignment and ordered orientation for the integration of optoelectronic devices. Here, our solution-processing approach employs capillary bridges to control the dewetting dynamics and confine the crystallization in the assembly of non-toxic Cs_2_AgBiBr_6_ microwire arrays. We demonstrate the strict alignment, high crystallinity, eliminated grain boundary, and ordered orientation of these as-prepared single-crystalline, double-perovskite microwire arrays. Based on these high-quality microwire arrays, we fabricate high-performance photodetectors with a responsivity of 1,625 A W^−1^, on/off ratio of 10^4^, and fast response speed of τ_decay_ = 0.04 ms and τ_rise_ = 0.28 ms. The long-term crystallographic and spectroscopic stability of Cs_2_AgBiBr_6_ microwire arrays has also been demonstrated through the 1 month exposure to air conditioning. Our strategy provides a new perception to fabricate stable perovskite microarrays for the integration of non-toxic optoelectronic devices.

## Introduction

Solution-processed lead halide perovskites APbX_3_ [A = cesium (Cs), methylammonium (MA), and formamidinium (FA); X = Cl, Br, I] have emerged as competitive to conventional semiconductors due to their unique physical properties, including tunable bandgap, long carrier diffusion length, and high carrier mobility, leading to wide applications of laser, photovoltaic, and optoelectronic devices (Ahmadi et al., 2017; Chen et al., 2017; Gao et al., 2018; Chen Z. et al., 2019a,b; Ning and Gao, 2019; Yuan et al., 2019). However, the instability in long-term storage and the toxicity of lead ion are two bottlenecks of lead halide perovskite in commercial applications (Schade et al., 2018; Igbari et al., 2019). Additionally, the instability mainly includes decomposition at high temperature and hydrolysis at high humidity. To improve the heat stability of perovskites, increasing the stoichiometric ratio of Cs^+^, and employing Dion-Jacobson layered perovskites are two common strategies. To achieve moisture stability, several strategies have been developed, including surface modifications by hydrophobic molecules and polymers and intercalations by fluorine-substituted amines (Chen G. S. et al., 2019; Chen et al., 2020). For reducing the toxicity of perovskites, lead-free halide perovskites have attracted broad attention via the substitution of Pb by introducing other low-toxicity elements, such as Tin (Sn) and germanium (Ge) (Ning et al., 2018). Considering that Sn^2+^/Ge^2+^-based perovskites are sensitive to oxygen and water owing to their active chemical properties, lead-free halide double perovskites could provide an alternative to realize the high-performance optoelectronic applications due to their high stabilities of heat, light, and moisture (Chu et al., 2019; Li et al., 2019; Ning et al., 2019; Muhammad and Yan, 2020).

Lead-free halide double perovskites with tunable bandgap spanning visible to near-infrared spectra and low carrier effective mass have a general formula of A_2_M^+^M'^3+^X_6_, where A^+^ and M^+^ are monovalent cations, M'^3+^ is a trivalent cation, and X^−^ is a halide ion (Slavney et al., 2016; Dai et al., 2020). As a representative double-perovskite, single-crystalline perovskite Cs_2_AgBiBr_6_ offers a substantial potential for not only high-efficiency photovoltaics but also multi-functional applications (Greul et al., 2017; Steele et al., 2018; Wu et al., 2018). For example, Tang and his colleagues have demonstrated sensitive X-ray detectors based on the Cs_2_AgBiBr_6_ bulk single crystal due to its high attenuation coefficient and higher heat stability (Pan et al., 2017; Yang et al., 2019). The Cs_2_AgBiBr_6_ thin film was fabricated toward a humidity sensor due to its extraordinary humidity-dependent electrical properties and high stability (Weng et al., 2019). Compared to the polycrystalline film and bulk single crystal, patterned single crystal micro-/nanostructures are more implemental for one-chip integrated devices. Therefore, it remains challenging in the fabrication of large-area single-crystalline Cs_2_AgBiBr_6_ micro/nanostructure arrays with precise spatial alignment, defined size and geometry, straight boundary, long-range order, pure orientation, and long-term stability.

In this report, we demonstrate the fabrication of lead-free perovskite Cs_2_AgBiBr_6_ microwire arrays via a capillary-bridge-mediated assembly method. A micropillar-structured template with asymmetric wettability of superhydrophilic tops and hydrophobic sidewalls is introduced to guide the formation of capillary bridges and direct dewetting process. These capillary bridges guarantee the controllable crystallization of Cs_2_AgBiBr_6_ microwires with regular positioning and strict spatial confinement, yielding perovskite microwire arrays with precise alignment, ordered orientation, and high crystallinity. The high crystalline quality and preferential (100) crystallographic orientation of Cs_2_AgBiBr_6_ microwire arrays are verified by selected area electron diffraction (SAED) and X-ray diffraction (XRD). Compared to the polycrystalline film, Cs_2_AgBiBr_6_ microwire arrays with high crystalline quality exhibit higher optoelectronic responsiveness because of their low defect density and suppressed grain boundary. Based on these single-crystalline, double-perovskite microwires, high-performing photodetectors were demonstrated with a high responsivity of 1,625 A W^−1^, a high light on/off ratio of 10^4^, a short decay time of 0.04 ms, and the rise time of 0.28 ms. We also demonstrate the essentially invariable crystallinity and absorption spectrum of Cs_2_AgBiBr_6_ single-crystal microwire arrays after 1 month exposure to air conditioning to evaluate their long-term stability.

## Experiment

### FAS Modification of the Template

The micropillar-structured template was firstly cleaned by ethanol, acetone, and isopropanol then treated with oxygen plasma for 10 min to keep its tops in the superhydrophilic state. Then, a glass with photoresist thin film was contacted with template tops for 30 s. After removing the glass, a drop of FAS and the template were placed in a vacuum dryer heating at 120°C for 6 h. When the dryer cooled down, FAS was successfully modified on the sidewalls of the template. The photoresist onto the tops of the template can be rinsed by acetone without destroying the FAS on the sidewalls.

### Preparation of Cs_2_AgBiBr_6_ Microwire Arrays

The pure-phase Cs_2_AgBiBr_6_ double-perovskite microwire arrays with high crystallinity, long-term stability, and suppressed grain boundary were prepared by a capillary-bridge-mediated assembly method which refers to a dewetting process guided by the micropillar-structured template with asymmetric wettability. The asymmetric wettability of the template was achieved by selectively modifying the FAS on the sidewalls of micropillars to realize their hydrophobic state. The Cs_2_AgBiBr_6_ precursor was prepared by adding stoichiometric CsBr, BiBr_3_, and AgBr into a DMSO solution within a glass bottle. A drop of precursor (15 mg mL^−1^, 10 μL) was injected onto the template and then covered with a flat substrate. After that, the system was heated at 80°C for 12 h in a vacuum oven. The Cs_2_AgBiBr_6_ microwire arrays were fabricated on the target substrate after the total evaporation of DMSO.

### Characterizations

The SEM (Hitachi 8010) at an accelerating voltage of 5 kV and AFM (Bruker Nano Inc. ICON2-SYS) was used to characterize the morphology of the Cs_2_AgBiBr_6_ double-perovskite microwire. The SAED pattern and TEM image were obtained by JEM-2100F operated at 100 kV to investigate the crystalline nature. Bruker D8 focus diffractometer equipped with Cu Kα radiation (λ = 1.5406 Å) was used to perform the XRD to evaluate the pure phase of those microwires. The PL and UV-vis absorption spectra were performed by Edinburgh Instruments FLS 1000 (UK) and Varian Cary 5000, respectively. The wettability of the template after FAS modification was characterized by measuring the contact angle (CA) using water on a Dataphysics OCA20 contact-angle system at ambient temperature. The average CA was obtained by measuring at 10 different positions on the same sample.

### Photodetector Fabrication

To fabricate the photodetector, we prepared highly crystalline Cs_2_AgBiBr_6_ double-perovskite microwire arrays on the 300 nm SiO_2_/Si substrates, which were cleaned with water, isopropanol, acetone, and ethanol. The electrodes (10 nm Cr and 100 nm Au) were directly evaporated on Cs_2_AgBiBr_6_ microwires using a shadow mask. The photoelectronic performance of the device was measured by Keithley 4200. The light irradiance of a light-emitting diode (LED, 455 nm) was tuned and mediated by a LED controller (Thorlabs DC2200) and silicon photodiode (Thorlabs, S130C), respectively.

## Results and Discussion

**Figure 1:**
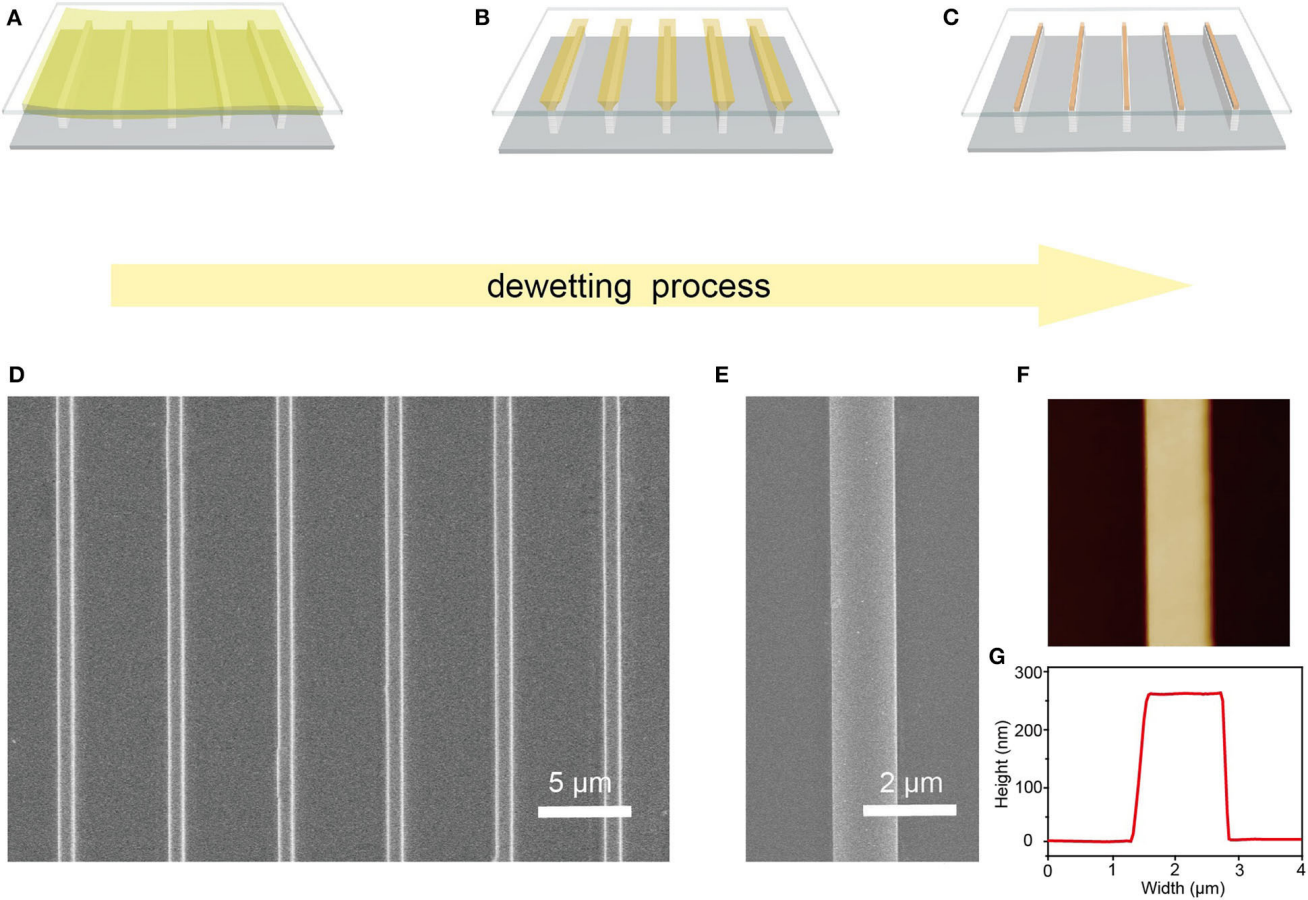
Fabrication process and morphology characterization of Cs_2_AgBiBr_6_ microwire arrays. **(A–C)** Schematic illustration of the dewetting and crystallization process confined in capillary bridges. **(D)** SEM image of as-prepared Cs_2_AgBiBr_6_ microwire arrays. **(E)** Zoom-in SEM image of a single Cs_2_AgBiBr_6_ microwire. **(F)** AFM image of a single Cs_2_AgBiBr_6_ microwire showing its smooth surface and **(G)** its width and height.

High-quality Cs_2_AgBiBr_6_ single-crystal microwire arrays were prepared through a capillary-bridge-mediated assembly method. The fabrication process of Cs_2_AgBiBr_6_ microwire arrays is schematically shown in Figures 1A–C. A sandwich-liked system, including a flat substrate (such as Si and glass), a periodic line-shape micropillar-structured template with superhydrophilic tops and hydrophobic sidewalls, and a drop of Cs_2_AgBiBr_6_ solution, is introduced to guide the dewetting process to yield pure orientated and high crystalline microwires. The micropillar-structured template was characterized by SEM (Supplementary Figure 1). The hydrophobic state of the sidewalls was realized by selectively modifying the FAS molecules. The schematic illustration FAS modification and wettability characterization are illustrated in Supplementary Figures 2, 3. The CA of the top is nearly 0°, showing a superhydrophilic state. The CA of the sidewall modified with FAS is about 120 ± 2°, demonstrating a hydrophobic state. A droplet of double-perovskite precursor was injected onto the asymmetric wettability template and then covered with a flat substrate to fabricate the Cs_2_AgBiBr_6_ microwire arrays. An entire liquid film was firstly formed between top surfaces of micropillars and the flat substrate because of the high adhesion of superhydrophilic tops. The gaps of the sidewalls were full of air, and no solution existed in those gaps. With the evaporation of the solvent, the continuous liquid film was separated into discrete line-shape capillary bridges pinned between superhydrophilic tops of the template and the substrate. Capillary bridges generated during the dewetting process could control the nucleation positions and direct the growth direction of the aimed high-quality microwire array. Capillary bridges shrunk and the concentrate of Cs_2_AgBiBr_6_ solution increased with the dewetting process going on. Once the solution saturated in capillary bridges, Cs_2_AgBiBr_6_ crystals would nucleate and constantly grow at the site pinned between micropillar tops and the target substrate. Finally, when the evaporation of the solvents completed, strict-aligned arrays were fabricated onto the target substrate.

Scanning electron microscopy (SEM) and atomic force microscopy (AFM) were carried out to characterize the crystallinity and surface morphology of the Cs_2_AgBiBr_6_ microwires. As Figure 1D shows, Cs_2_AgBiBr_6_ microwires have regular alignment and precise position. The zoom-in SEM image (Figure 1E) illustrates the microwire has a smooth surface and no visible grain boundary, indicating the high crystalline nature of these microwires. The AFM image (Figure 1F) shows an individual microwire with a smooth surface, a width of 1.5 μm, and a height of 260 nm (Figure 1G), which further demonstrates the high crystallinity of the microwire.

**Figure 2:**
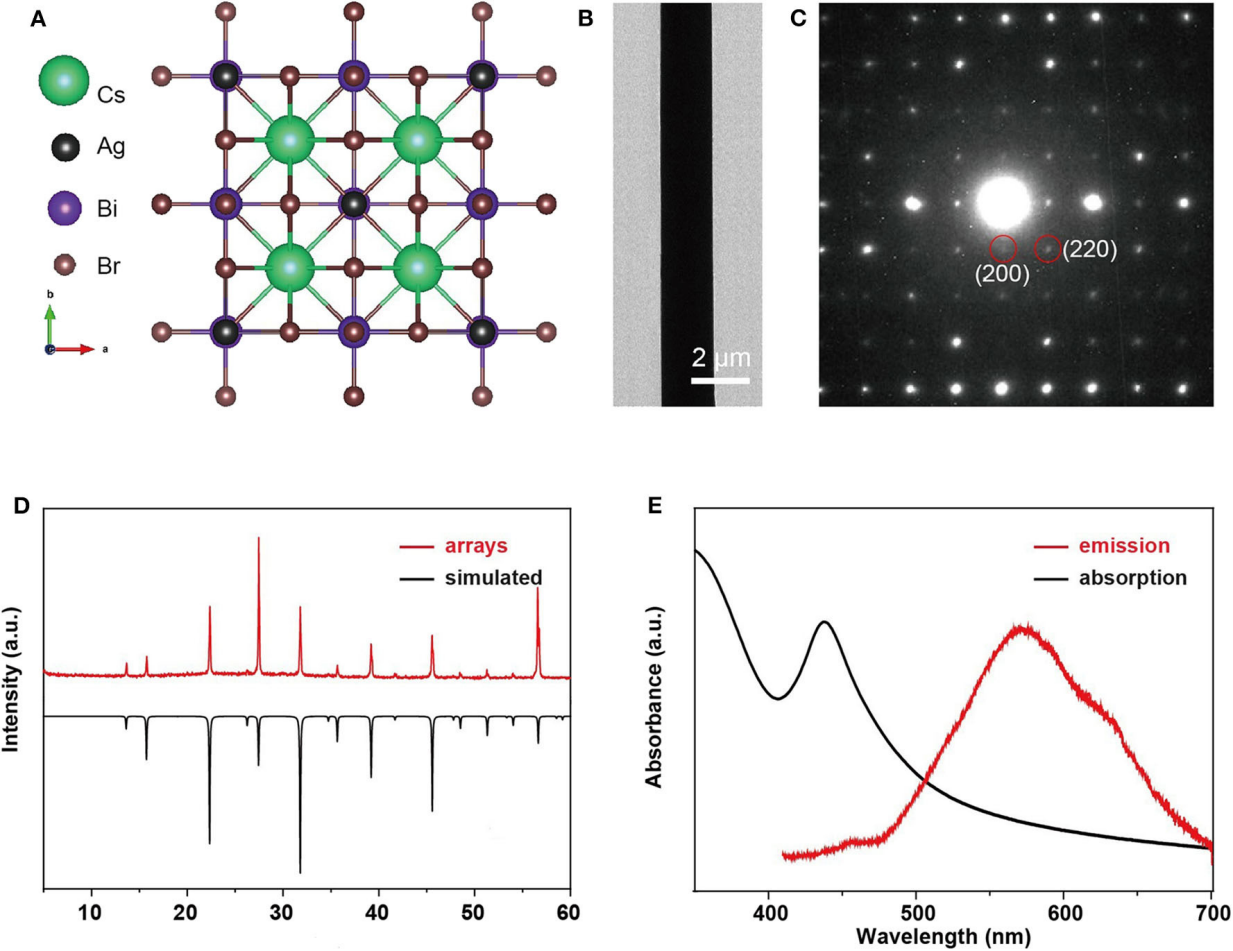
Crystallographic and spectroscopic characterizations of single-crystalline, double-perovskite microwire arrays. **(A)** Crystal structure of Cs_2_AgBiBr_6_ single crystal viewed from [001] directions. **(B)** TEM image and **(C)** SAED pattern of a Cs_2_AgBiBr_6_ single-crystal microwire. **(D)** XRD pattern of Cs_2_AgBiBr_6_ microwire arrays compared with the corresponding simulation. **(E)** UV-vis absorption and PL emission spectra of Cs_2_AgBiBr_6_ microwire arrays.

The transmission electron microscope (TEM), SAED, and XRD measurements are performed to investigate the crystal structure and crystallographic orientation of as-prepared Cs_2_AgBiBr_6_ arrays. We failed to record the high-resolution transmission electron microscope image (HRTEM) because of the easy decomposition of Cs_2_AgBiBr_6_ under strong electron beam radiation. UV-vis absorbance and photoluminescence (PL) spectra were performed at room temperature. The crystal structure of Cs_2_AgBiBr_6_ is depicted in Figure 2A (Yang et al., 2019). The TEM image (Figure 2B) illustrated that the microwire has a homogeneous surface and perfect morphology. The SAED pattern (Figure 2C) shows clear diffraction spots, demonstrating the good single crystal nature and the pure crystallographic orientation along [001]. As Figure 2D shows, the XRD pattern with sharp peak confirms the pure phase of Cs_2_AgBiBr_6_ (*a* = *b* = *c* = 11.27Å, α = β = γ = 90°, space group *Fm-3m*) without any other impurity, matching well with the results of the simulation. The absorption peak of the microwire arrays is at 438 nm, and the emission peak is at 570 nm (Figure 2E), which is consistent with a previous report (Pan et al., 2017).

**Figure 3:**
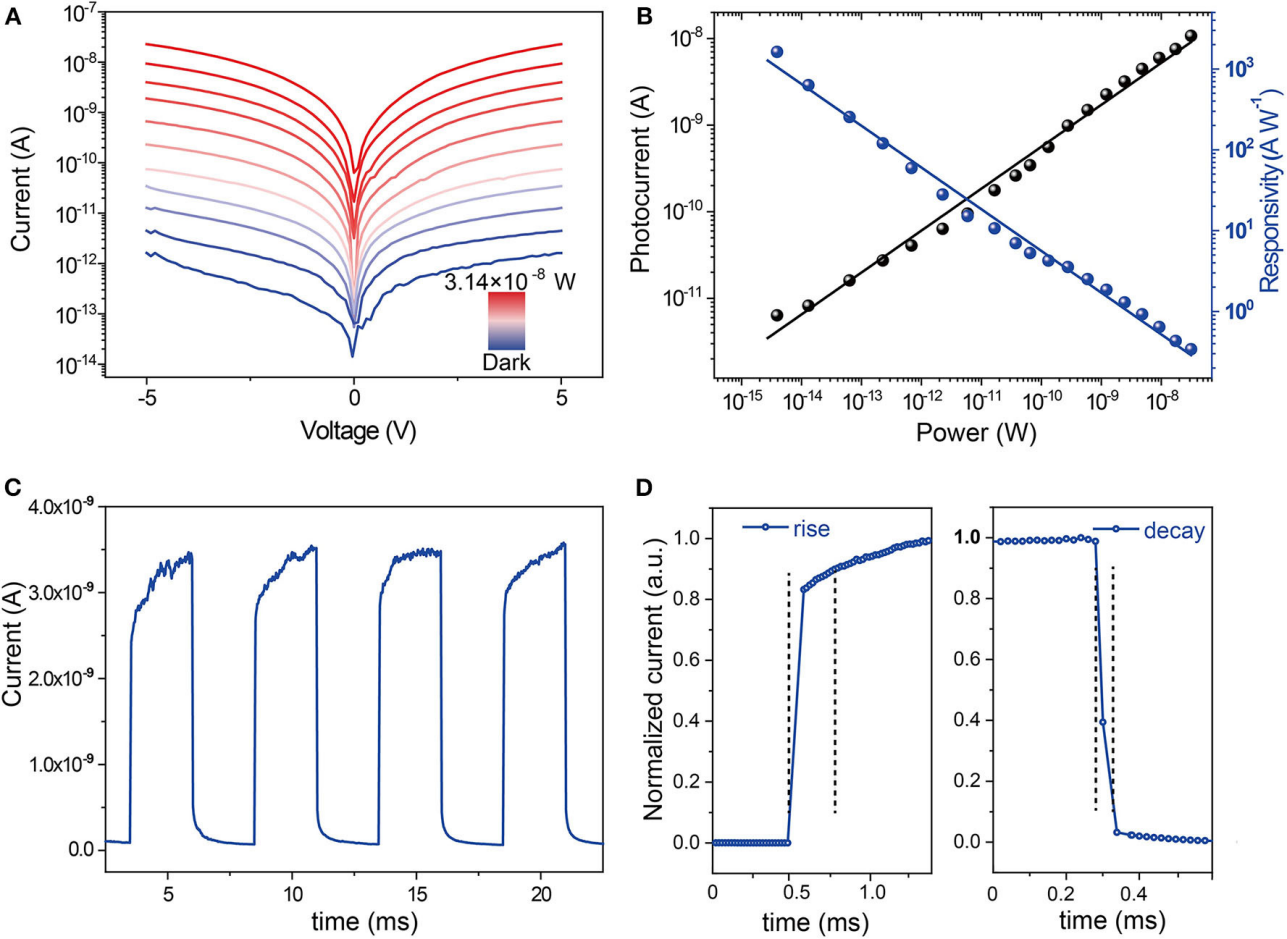
Performances of the photodetector based on single-crystalline Cs_2_AgBiBr_6_ microwire arrays. **(A)** *I-V* curves of the device under different illumination intensities and dark conditions. **(B)** The photocurrent and responsivity of the device under different illumination power. **(C)** *I–t* response with four on-off cycles and **(D)** temporal response of the device.

Then we investigated the performance of the fabricated detectors based on Cs_2_AgBiBr_6_ microwire arrays. The Cs_2_AgBiBr_6_ microwires were fabricated on SiO_2_/Si substrate, and Cr/Au (10/100 nm) electrodes were evaporated with shadow masks. The morphology of the device was characterized by SEM. A typical SEM image exhibits the structure of a photodetector based on several parallel Cs_2_AgBiBr_6_ microwires (Supplementary Figure 4). A series of *I–V* characteristic curves of Cs_2_AgBiBr_6_ arrays are displayed in Figure 3A, which were measured under dark conditions and 455 nm light illumination with different irradiation power by scanning bias from −5 to 5 V. The photodetector exhibits a current of 1.5 × 10^−8^ A with an irradiation intensity of 3.14 × 10^−8^ W and a dark current of 6.02 pA under the bias of 5 V, and thus, a light on–off ratio of 10^4^ is calculated. The photocurrent increased with increasing irradiation power intensity, which was measured by *I*_ph_ = *I*_light_–*I*_dark_, where *I*_light_ and *I*_dark_ are currents under light illumination and dark, respectively. The responsivity (*R*) of the detector was calculated by *R* = *I*_ph_/*P*, in which *P* is the incident light power. The responsivity reached 1,625 A W^−1^ under the irradiation intensity of 3.92 × 10^−15^ W. As Figure 3B shows, the photocurrent manifests a positive linear relationship with the illumination power, while the responsivity exhibits a negative linear relationship with the illumination power. The temporal photoresponse of the device was measured by periodically switching the light source with an intensity of 3.14 × 10^−8^ W, which is presented in Figure 3C, which suggests that the device has an excellent response toward the light state between on and off. The response time is defined as the current increase from 10 to 90% of the maximum for rise, and vice versa for decay. As shown in Figure 3D, the rise time is 0.28 ms and the decay time is 0.04 ms, indicating a fast response speed. The microwire with suppressed grain boundaries and high crystallinity is beneficial to the realization of high-performance optoelectronic devices. To verify the advantage of Cs_2_AgBiBr_6_ microwire arrays, we performed *I–V* measurements of the device based on the spin-coated polycrystalline thin film. The SEM image of the thin film was displayed in Supplementary Figure 5. The current of the thin film with the irradiation intensity of 3.14 × 10^−8^ W and under the bias of 5 V is 3.1 × 10^−10^ A (Supplementary Figure 6), which is less than that of microwire arrays, while the dark current of the film is a little higher than that of microwire arrays. The responsivity of the photodetector based on the Cs_2_AgBiBr_6_ thin film was only 0.68 A W^−1^, which is three orders of magnitude lower than that of Cs_2_AgBiBr_6_ microwire arrays. The high-efficiency photocarrier generation attribute to the high crystalline nature and suppressed grain boundaries of Cs_2_AgBiBr_6_ microwire arrays, thus leading to higher performance of photodetectors based on microwire arrays.

**Figure 4:**
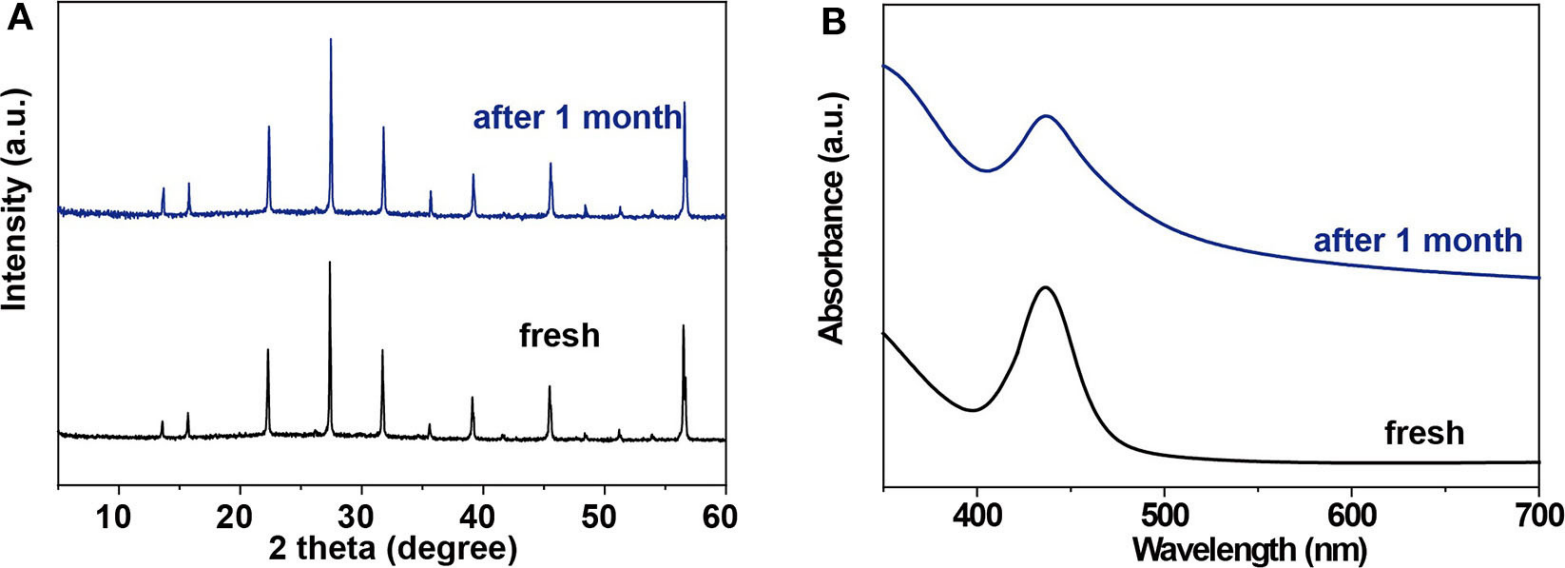
Stability of single-crystalline Cs_2_AgBiBr_6_ microwire arrays. **(A)** XRD patterns and **(B)** absorption spectra of Cs_2_AgBiBr_6_ microwire arrays before and after the storage at room temperature (22°C) and relative humidity of 30% in air conditioning, respectively.

It has been demonstrated that the bulk Cs_2_AgBiBr_6_ single crystal has good resistance to heat treatment and humid atmosphere (Ning et al., 2019). Our microwires are expected to have good stability too. To assess the stability of Cs_2_AgBiBr_6_ microwire arrays, we fabricated microwire arrays on a Si substrate and kept it under ambient conditions for 30 days. We measured XRD and absorption spectra to verify their stability. As shown in Figures 4A,B, the XRD pattern and absorption curve keep consistent with initial characterizations, suggesting that Cs_2_AgBiBr_6_ microwire arrays have excellent stability in ambient conditions.

## Conclusion

We employ the capillary-bridge-assisted method to prepare Cs_2_AgBiBr_6_ microwire arrays with a smooth surface, pure orientation, and ordered alignment, then demonstrating non-toxic blue-light photodetectors with high performance and long-term stability. Capillary bridges control the fluid dynamics in the dewetting process, thus leading to the oriented growth of Cs_2_AgBiBr_6_ microwire arrays. Compared to the Cs_2_AgBiBr_6_ polycrystalline film with a large grain boundary, as-prepared microwire arrays have large grain sizes and eliminated grain boundaries, which are beneficial for improving the performance of devices. Photodetectors based on double-perovskite microwire arrays exhibit a high responsivity of 1,625 A W^−1^, on-off ratio exceeding 10^4^, and fast response speed. We believe that our work provides a new alternative for the integration of non-toxic perovskite photodetectors based on perovskite single-crystal arrays.

## Data Availability Statement

The raw data supporting the conclusions of this article will be made available by the authors, without undue reservation.

## Author Contributions

YP, JZ, and YZ contributed equally to this manuscript. YP, JZ, and HG co-wrote the manuscript. YZ performed the experiments. JF, HG, YW, and LJ designed the project. HG, CZ, YW, and LJ discussed the results part and revised the manuscript. All authors approved the final version of the manuscript.

## Conflict of Interest

The authors declare that the research was conducted in the absence of any commercial or financial relationships that could be construed as a potential conflict of interest. The reviewer QY declared a shared affiliation, though no other collaboration, with the authors YP, CZ to the handling editor.
