# Supplementary material for: Capillary-Bridge Controlled Patterning of Stable Double-Perovskite Microwire Arrays for Non-toxic Photodetectors
Source: Front Chem. 2020 Aug 25;8:632. doi: 10.3389/fchem.2020.00632 (PMC7477743; doi:10.3389/fchem.2020.00632)
Supplement: Supplementary file 1 [file Data_Sheet_1.docx]

Supplementary Material


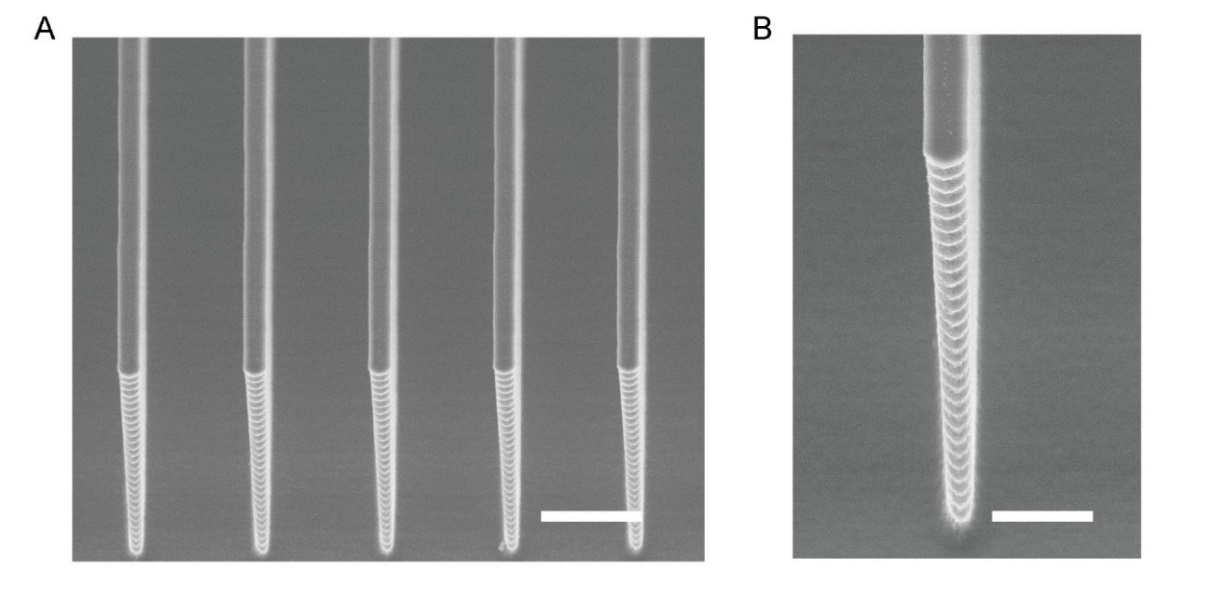


**Supplementary Figure 1.** Characterization of micropillar-structured template. (A) SEM image of the template. (B) Zoom in SEM image of the template. Scar bars: 5 μm.


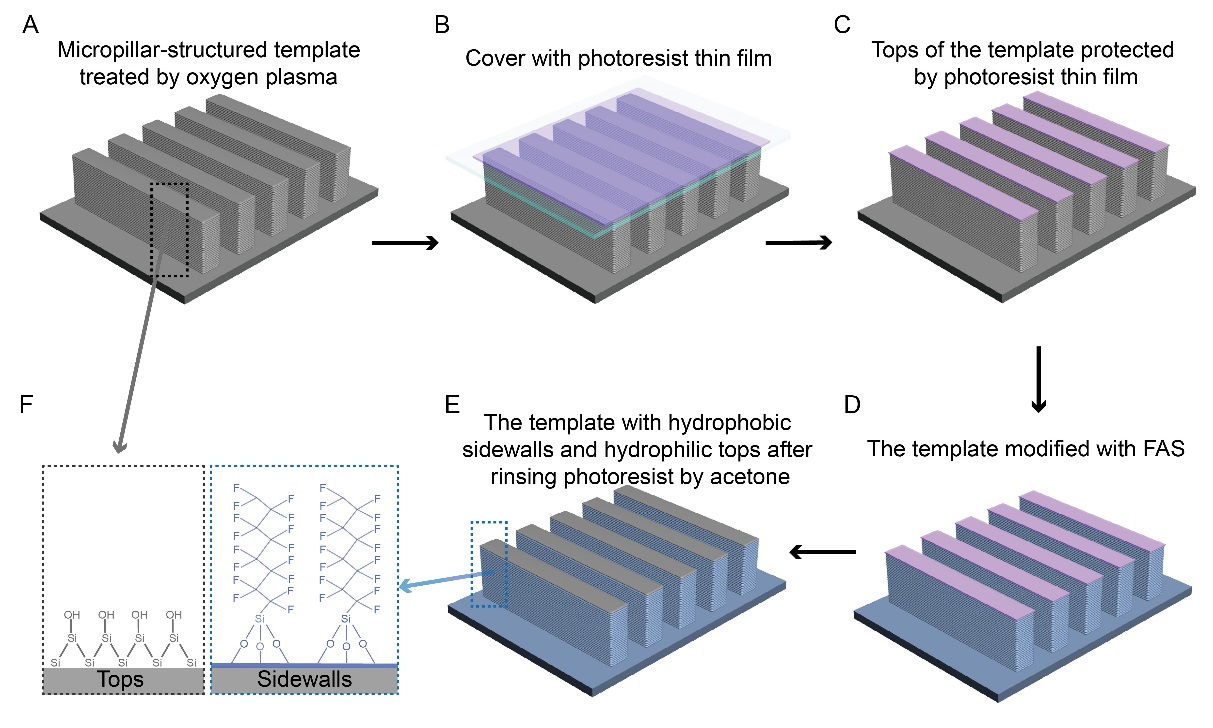


**Supplementary Figure 2**. Schematic illustration of FAS modification. (A) Micropillar-structured template after oxygen plasma treatment for 10 minutes. (B) Tops of template covered with a glass spin-coated with photoresist. (C) Tops of the template with a protect layer of photoresist thin film. (D) The template modified with FAS. (E) The template with hydrophobic sidewalls and superhydrophilic tops after immersing in acetone. (F) Schematic illustration of the superhydrophilic state in tops and hydrophobic state in sidewalls, respectively.

**
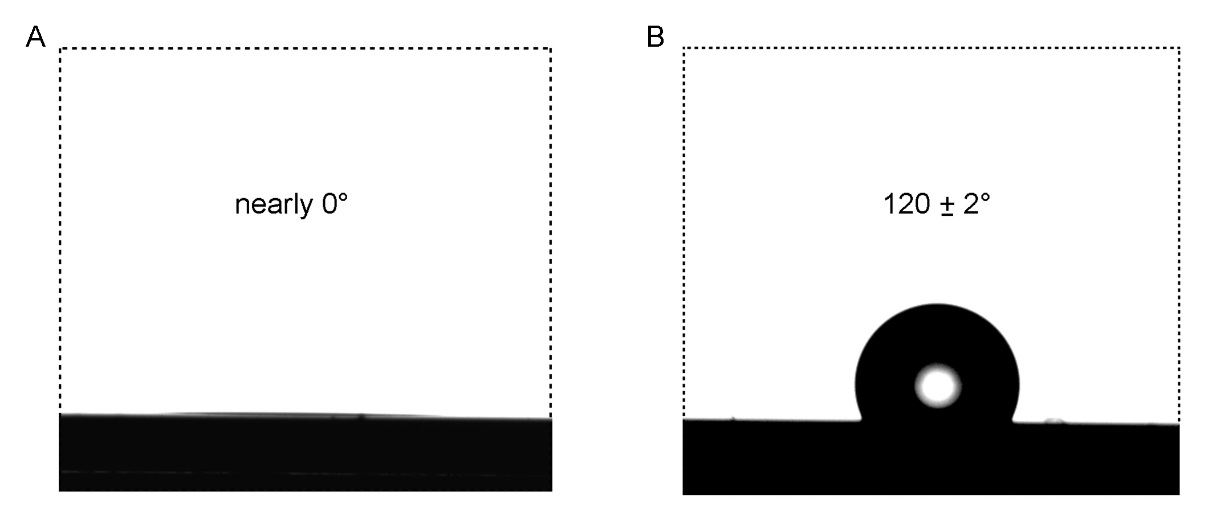
**

**Supplementary Figure 3.** CA characterization of the asymmetric wettability template. (A) The contact angle of water on the top, showing a superhydrophilic state with a contact angle of nearly 0°. (B) The contact angle of water on sidewalls modified with FAS, showing a contact angle of about 120 ± 2°, demonstrating a hydrophobic state.


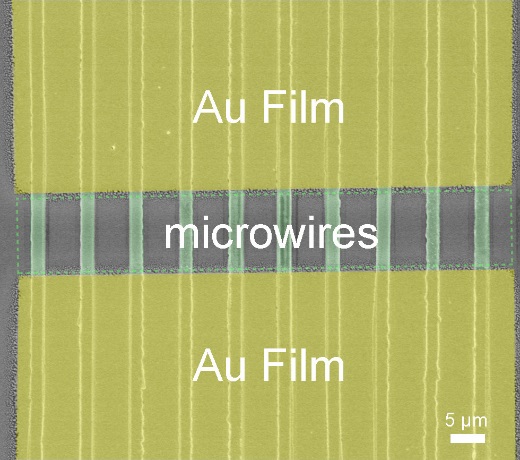


**Supplementary Figure 4.** Pseudocolor processed SEM image of the photodetector based on Cs_2_AgBiBr_6_ microwire arrays.


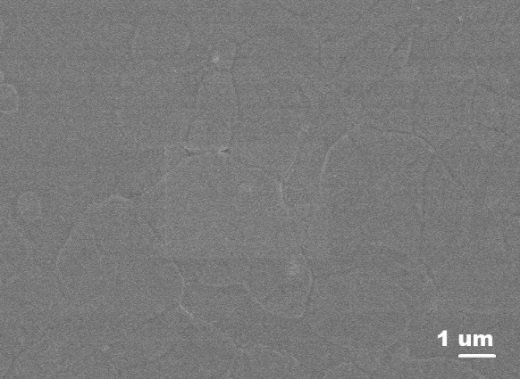


**Supplementary Figure 5.** SEM Characterization of Cs_2_AgBiBr_6_ film illustrating large grain size.


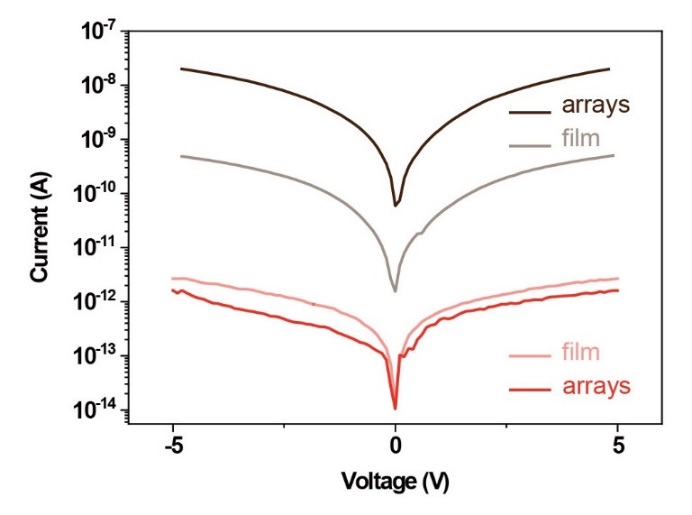


**Supplementary Figure 6.** Comparison of photodetector performance based on Cs_2_AgBiBr_6_ film and Cs_2_AgBiBr_6_ single crystal arrays. The photocurrent of the single crystal wire arrays is higher than thin film under the same irradiation intensity.
